# Changes in Nutrient Components and Digestive Enzymatic Inhibition Activities in Soy Leaves by Ethephon Treatment

**DOI:** 10.3390/plants12203640

**Published:** 2023-10-21

**Authors:** Ji Ho Lee, Du Yong Cho, Kyeong Jin Jang, Jong Bin Jeong, Ga Young Lee, Mu Yeun Jang, Ki Ho Son, Jin Hwan Lee, Hee Yul Lee, Kye Man Cho

**Affiliations:** 1 Department of Green Bio Science and Agri-Food Bio Convergence Institute, Gyeongsang National University, Jinju 52725, Republic of Koreasonkh@gnu.ac.kr (K.H.S.); 2 Department of Food Science, Gyeongsang National University, Naedongro 139-8, Jinju 52849, Republic of Korea; 3 Division of Horticultural Science, Gyeongsang National University, Jinju 52725, Republic of Korea; 4 Department of Life Resource Industry, Dong-A University, 37, Nakdong-daero 550 beon-gil, Saha-gu, Busan 49315, Republic of Korea; schem72@dau.ac.kr

**Keywords:** ethephon, soybean leaf, water-soluble vitamin, isoflavone, digestive enzymatic inhibition activity

## Abstract

In this study, the high isoflavone-enriched soy leaves (IESLs) were manufactured by treating with the chemical inducer ethephon, a plant growth regulator, to confirm changes in the properties of soy leaves (SLs), which are underutilized. Ethephon treatment concentrations consisted of 0 (SL1), 150 (SL2), and 300 (SL3) μg/mL. The composition analysis and physiological activity were conducted according to the ethephon treatment concentration of SLs. There was no significant difference in the proximate composition and fatty acids, except for an increase with increasing ethephon treatment concentrations. Depending on the ethephon treatment concentration, free amino acids increased to 1413.0, 1569.8, and 2100.4 mg/100 g, and water-soluble vitamins increased to 246.7, 244.7, and 501.6 mg/100 g. In particular, the functional substance isoflavone increased significantly to 1430.11, 7806.42, and 14,968.00 μg/g. Through this study, it was confirmed that the nutritional components and isoflavones of SLs increased according to the ethephon treatment concentration, a chemical inducer treatment agent. This can be used as a high-value-added biosubstance for raw materials for functional foods, cosmetics, and for natural drugs.

## 1. Introduction

Soybeans are such an important source of protein and fat that they are called “beef from the field” and are one of the world’s top three resources. The most characteristic component related to the physiological activity of soybeans is known as isoflavones. Soy isoflavones are also called “phytoestrogen” as phytochemicals, which are divided into glycoside (daidzin, glycitin, and genistin) with glucose and aglycone (daidzein, glycitein, and genistein) without glucose. Isoflavones have various physiological characteristics such as antitumor, anticancer, antioxidant, menopausal symptom relief, heart disease, diabetes, and osteoporosis prevention [1,2,3]. While soybeans are actively studied and used worldwide, soy leaves (SLs), a by-product of soybean production, are rarely consumed as food except in the Republic of Korea, meaning they are severely underutilized. SLs able to be mass-produced, but they only serve as a simple nutrient source, and the active metabolites that exist are insufficient. However, it has recently been reported that SLs contain various functional ingredients such as flavones, flavonols, terocapans, phenolic compounds, and sugar alcohols, in addition to isoflavones, anthocyanins, and saponins. SL extract has anti-obesity, anti-diabetes, antioxidant, and anti-inflammatory effects, so interest in the food value of SLs is increasing [4,5,6,7,8,9].

One of the ways to increase the functional substances within SLs is to treat them with plant growth regulators or chemical inducers. The ethylene that promotes ripening regulates many aspects of the plant’s life cycle, including seed germination, root initiation, flower development, fruit maturation, aging, and response to biological and nonbiological stress. Therefore, it is crucial in environmental responses affecting plant adaptation and reproductive suitability [10]. Ethylene is challenging to apply directly to plants because of its relatively low solubility. However, (2-Chloroethyl)phosphonic acid (ethephon), a synthetic growth regulator, can be used in a liquid state. Treatment with ethephon induces the release of ethylene, regulates the level and function of endogenous ethylene, increases glutathione content and antioxidant metabolism, and leads to stress elimination. Additionally, ethylene interacts with secondary metabolites, osmolytes, and carbohydrates and induces stress tolerance mechanisms in plants. The interaction of ethylene with nutrients and plant hormones helps promote plant growth by inducing plant metabolism [11,12]. Studies have reported that treating with ethylene and ethephon during the crop production stage of SLs activates a series of structural genes involved in isoflavonoid biosynthesis and improves the production of isoflavones [13].

There have been many studies on the changes and characteristics of crops under these various environmental conditions [14,15,16]. However, more research on the change in the SL composition according to the concentration of ethephon treatment is needed. The study of SLs according to the ethephon treatment concentration (ethephon 0, 150, and 300 μg/mL treatment, respectively SL1, SL2, and SL3) is expected to produce high isoflavone SLs with high utilization value efficiently and help manufacture high-value-added products. Therefore, in this study, changes in active ingredients and physiological activity were compared and evaluated SL treatment with increasing ethephon concentrations to increase the value of high isoflavone-enriched soy leaves (IESLs).

## 2. Results

### 2.1. Changes of Proximate Compositions in SLs by Ethephon Treatment

Proximate compositions were measured to confirm the change in the nutritional composition of SLs according to the ethephon treatment concentration, and the results are shown in Table 1. The main proximate compositions are protein and carbohydrates; the proteins are SL1 (20.6 g/100 g), SL2 (20.9 g/100 g), and SL3 (25.0 g/100 g), which increased with increasing ethephon treatment. In comparison, carbohydrates decreased to SL1 (57.2 g/100 g), SL2 (56.1 g/100 g), and SL3 (53.0 g/100 g). In addition, moisture (8.6 to 9.0 g/100 g), ash (8.8 to 10.0 g/100 g), and fat (3.3 to 5.3 g/100 g) did not change significantly. Overall, there was no significant difference in proximate compositions with increasing ethephon treatment.

### 2.2. Changes of Fatty Acid Contents in SLs by Ethephon Treatment

**Figure 1:**
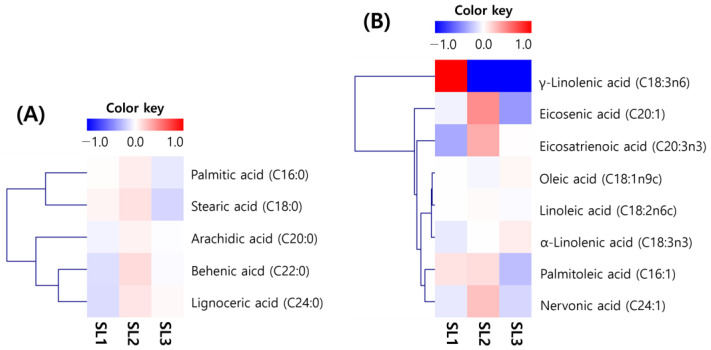
Heatmap for analysis of fatty acids in soy leaves by ethephon treatment. (**A**) Saturated fatty acid; and (**B**) unsaturated fatty acid. Ethephon treatments at 0 μg/mL, SL1; ethephon treatments at 150 μg/mL, SL2; ethephon treatments at 300 μg/mL, SL3.

The changes in fatty acids in SLs according to the ethephon treatment concentration are shown in Figure 1 and Supplementary Table S1. No distinct changes in fatty acids could be identified in the heatmap. Most fatty acids tended to increase and decrease according to the ethephon treatment concentration, and only α-linolenic acid increased proportionally to the treatment concentration. Compared to other fatty acids, γ-linolenic acid had a distinct color change. Still, it was relatively distinct because it was not detected in the ethephon treatment sample (Figure 1). Overall, the total fatty acid content was SL1 (976.9 mg/100 g), SL2 (1037.7 mg/100 g), and SL3 (996.7 mg/100 g). There was no significant difference in treatment concentration. Palmitic acid, which had a high proportion among saturated fatty acids, decreased after slightly increasing to SL1 (212.3 mg/100 g), SL2 (224.8 mg/100 g), and SL3 (191.2 mg/100 g). In unsaturated fatty acids, α-linolenic acid increased with treatment concentration to SL1 (383.8 mg/100 g), SL2 (419.6 mg/100 g), and SL3 (447.1 mg/100 g) (Supplementary Table S1).

### 2.3. Changes of Free Amino Acid Contents in SLs by Ethephon Treatment

**Figure 2:**
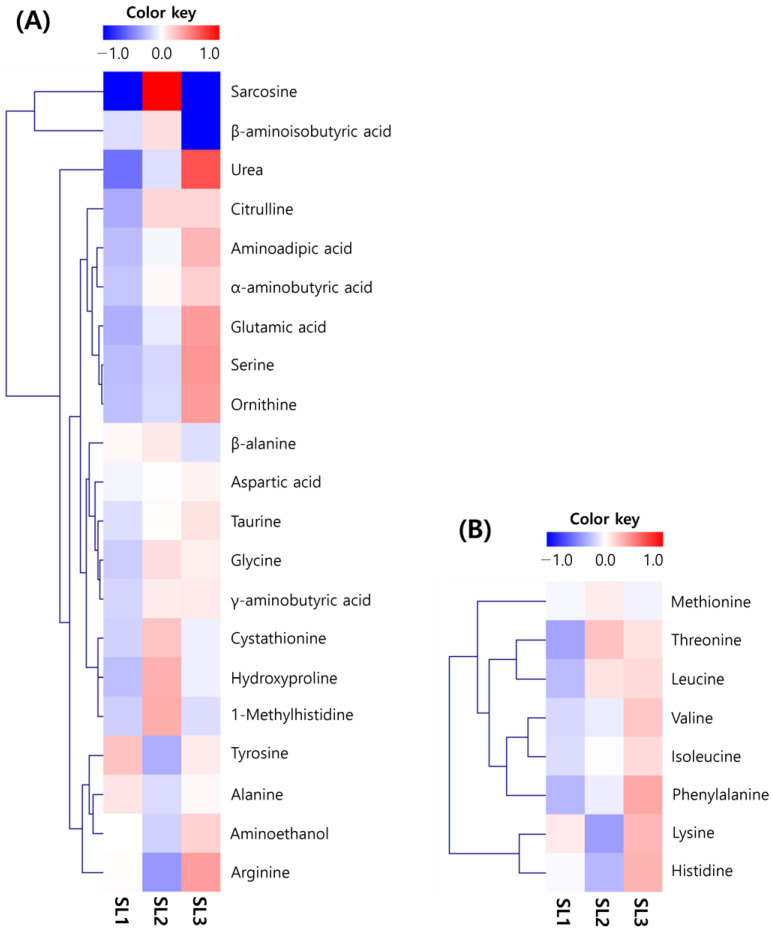
Heatmap for analysis of free amino acids in soybean leaves by ethephon treatment. (**A**) non-essential amino acid; and (**B**) essential amino acid. Ethephon treatments at 0 μg/mL, SL1; ethephon treatments at 150 μg/mL, SL2; ethephon treatments at 300 μg/mL, SL3.

The patterns of the change in the free amino acid content in SLs, according to the ethephon treatment concentration as a heatmap, vary (Figure 2). Many patterns increased according to the treatment concentration, which was higher in content than other amino acids. Among the nonessential amino acids (NEAA), there was a 4-fold increase for urea (79.4 to 304.4 mg/100 g), about 2 fold for glutamic acid (65.8 to 132.8 mg/100 g), and about 2 fold for serine (69.2 to 132.8 mg/100 g); there was only a slight change for aspartic acid (174.2 to 189.6 mg/100 g) and γ-aminobutyric acid (141.0 to 182.6 mg/100 g). Among the essential amino acids (EAA), hydrophobic amino acids tended to increase, and in particular, phenylalanine (84.0 to 157.0 mg/100 g) increased about 2 fold. The total amino acids were SL1 (1413.0 mg/100 g), SL2 (1569.8 mg/100 g), and SL3 (2100.4 mg/100 g), and both EAA and NEAA increased with increasing ethephon treatment concentration (Supplementary Table S2).

### 2.4. Changes of Water-Soluble Vitamin Contents in SLs by Ethephon Treatment

**Figure 3:**
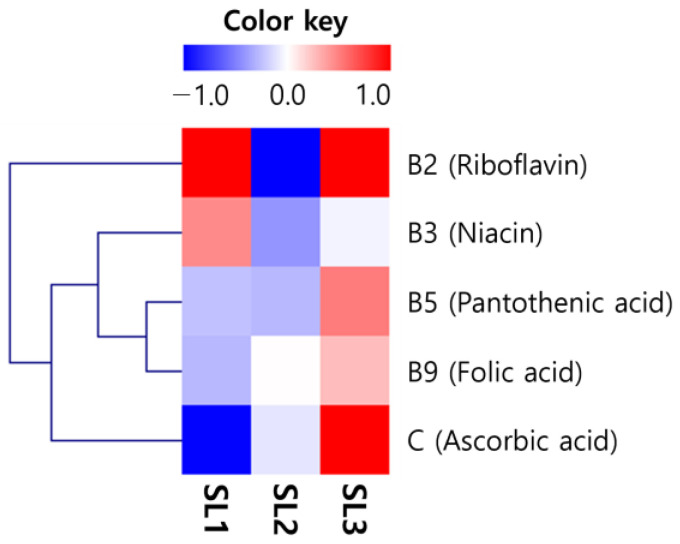
Heatmap for analysis of water-soluble vitamins in soy leaves by ethephon treatment. Ethephon treatments at 0 μg/mL, SL1; ethephon treatments at 150 μg/mL, SL2; ethephon treatments at 300 μg/mL, SL3.

The SL water-soluble vitamin contents increased according to the ethephon treatment concentration, except for riboflavin and niacin (Figure 3). In particular, pantothenic acid, which had the highest proportional increase, increased 2 fold to SL1 (215.7 mg/100 g), SL2 (206.0 mg/100 g), and SL3 (431.0 mg/100 g). Ascorbic acid, which increased the most significantly compared to untreated, increased by about 200 fold to SL1 (0.2 mg/100 g), SL2 (17.6 mg/100 g), and SL3 (40.8 mg/100 g). The total content increased significantly after a slight decrease to SL1 (246.7 mg/100 g), SL2 (244.7 mg/100 g), and SL3 (501.6 mg/100 g) (Table 2).

### 2.5. Changes of TP and TF Contents in SLs by Ethephon Treatment

**Figure 4:**
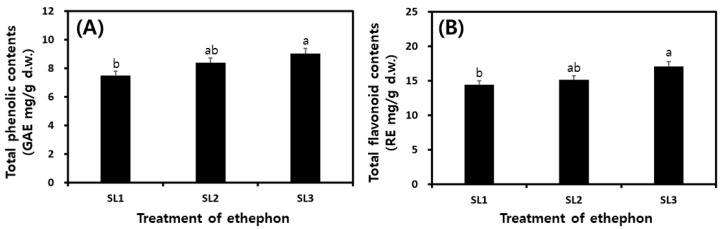
Comparison of total phenolic and total flavonoid contents in soy leaves by ethephon treatment. (**A**) Total phenolic contents; and (**B**) total flavonoid contents. All values are means of determination in three independent experiments. Different small letters correspond to the significant differences relating to the treatment concentration using Tukey’s multiple test (*p* < 0.05). Ethephon treatments at 0 μg/mL, SL1; ethephon treatments at 150 μg/mL, SL2; ethephon treatments at 300 μg/mL, SL3.

The total phenolic (TP) and total flavonoid (TF) contents of SLs were measured according to the ethephon treatment concentration, and the results are shown in Figure 4. The TP contents result is SL1 7.50 GAE mg/g, SL2 8.38 GAE mg/g, SL3 9.01 GAE mg/g (Figure 4A), and TF contents is SL1 14.41 RE mg/g, SL2 15.12 RE mg/g, and SL3 17.10 RE mg/g (Figure 4B). TP and TF contents increased with increasing ethephon concentration.

### 2.6. Changes of Isoflavones Contents in SLs by Ethephon Treatment

**Figure 5:**
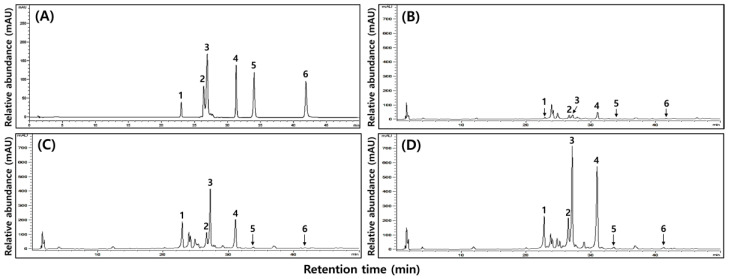
Typical isoflavone HPLC chromatogram of the 50% methanol extracts in soy leaves by ethephon treatment. (**A**) Standard; (**B**) treatment concentration of ethephon: 0 μg/mL; (**C**) treatment concentration of ethephon: 150 μg/mL; and (**D**) treatment concentration of ethephon: 300 μg/mL. One, Daidzin; 2, Genistin; 3, Manloyldaidzin; 4, Manloylgenistin; 5, Daidzein; and 6, Genistein.

**Figure 6:**
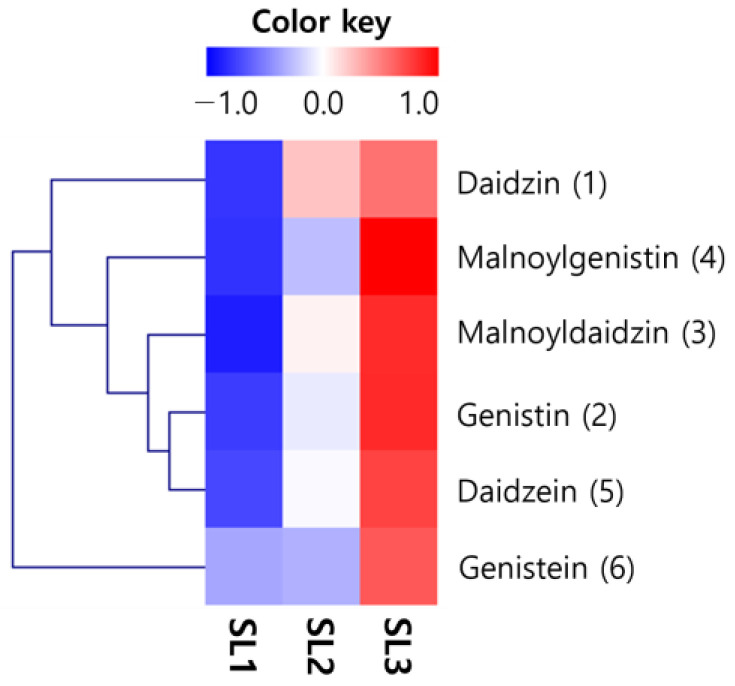
Heatmap for analysis of isoflavones in soy leaves by ethephon treatment. Ethephon treatments at 0 μg/mL, SL1; ethephon treatments at 150 μg/mL, SL2; ethephon treatments at 300 μg/mL, SL3.

As a result of isoflavone analysis, HPLC identified four types of glycosides (daidzin, genistin, malonyldaidzin, and malonylgenistin) and two types of aglycones (daidzein and genistein) identified within SLs (Figure 5). The change in content according to the ethephon treatment concentration was visualized as a heatmap. An apparent change was confirmed (Figure 6). All components increased with the treatment concentration, and the increase was significant, except for genistein, with the presence or absence of ethephon treatment. The isoflavone type, which had the most significant proportional increase, increased by about 12 fold for malonylglycosides (859.20 to 10,911.73 μg/g). Glycosides increased by approximately 7 fold (495.18 to 3740.54 μg/g) and aglycons (75.73 to 315.73 μg/g) by about 4 fold. The total isoflavone contents increased by approximately 10 fold to SL1 (1430.11 μg/g), SL2 (7806.42 μg/g), and SL3 (14,968.00 μg/g), confirming a distinct increase depending on the ethephon treatment concentration (Table 3).

### 2.7. Changes of Antioxidant Activities in SLs by Ethephon Treatment

**Figure 7:**
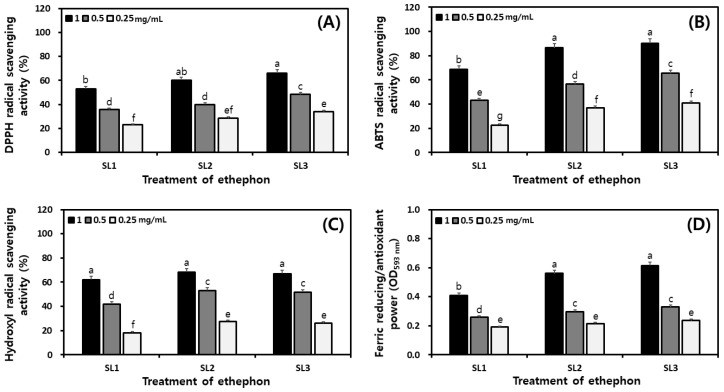
Comparison of the antioxidant activities in soy leaves by ethephon treatment. (**A**) DPPH radical scavenging activity; (**B**) ABTS radical scavenging activity; (**C**) hydroxyl radical scavenging activity; and (**D**) ferric reducing/antioxidant power. All values are present as the mean ± SD of pentaplicate determination. Different small letters correspond to the significant differences relating to the treatment concentration using Tukey’s multiple test (*p* < 0.05). Ethephon treatments at 0 μg/mL, SL1; ethephon treatments at 150 μg/mL, SL2; ethephon treatments at 300 μg/mL, SL3.

The antioxidant activity according to the ethephon treatment concentration was measured and shown in Figure 7. The sample extracts were measured by diluting at 1, 0.5, and 0.25 mg/mL concentrations. In the sample extract at a concentration of 1 mg/mL, the 2,2-diphenyl-1-picrylhydrazyl (DPPH) radical scavenging activity was SL1, SL2, and SL3 showed 52.97%, 60.19%, and 66.18%, respectively, and increased by 13.21% (Figure 7A). 2,2-azino-bis (3-ethylbenzothiazoline-6-sulfonic acid) (ABTS) radical scavenging activity was SL1, SL2, and SL3 showed 21.47%, 68.74%, and 86.50%, respectively, and increased by 21.47% (Figure 7B). Hydroxyl radical scavenging activity was SL1, SL2, and SL3 showed 62.21%, 68.36%, and 66.99%, respectively, decreased slightly after the increase and no significant differences (Figure 7C). Ferric reducing ability of plasma (FRAP) reducing force, SL1, SL2, and SL3 showed activity of 0.410, 0.561, and 0.615, respectively, increasing with the ethephon treatment concentration (Figure 7D). Overall, antioxidant activity increased with ethephon treatment concentration except for hydroxyl radical scavenging activity.

### 2.8. Changes of Digestive Enzyme Inhibitory Activities in SLs by Ethephon Treatment

**Figure 8:**
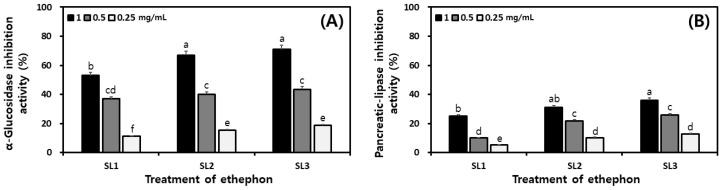
Comparison of the antioxidant and digestive enzymatic inhibition activities in soy leaves by ethephon treatment. (**A**) α–glucosidase inhibition activity; and (**B**) pancreatic lipase inhibition activity. All values are present as the mean ± SD of pentaplicate determination. Different small letters correspond to the significant differences relating to the treatment concentration using Tukey’s multiple test (*p* < 0.05). Ethephon treatments at 0 μg/mL, SL1; ethephon treatments at 150 μg/mL, SL2; ethephon treatments at 300 μg/mL, SL3.

The digestive enzyme inhibitory activity according to the ethephon treatment concentration was measured and shown in Figure 8. The sample extracts were measured by diluting at 1, 0.5, and 0.25 mg/mL concentrations. The α-glucosidase inhibitory activity showed activity of 53.03%, 67.01%, and 70.96%, respectively, when treated with 1.0 mg/mL in SL1, SL2, and SL3 (Figure 8A). Pancreatic lipase inhibitory activity showed 21.88%, 31.14%, and 36.02% (Figure 8B). Compared to the pancreatic lipase, α–glucosidase inhibitory activity was better, and both activities increased with ethephon treatment concentration.

## 3. Discussion

The fresh SLs (based on biomass) contain about 69.9 g/100 g of moisture, 6.6 g/100 g of protein, 0.3 g/100 g of fat, 2.2 g/100 g of ash, and 21.0 g/100 g of carbohydrates [17]. Nutritional components generally differed greatly from this study, and the results in the Food Composition Table were analyzed on a biomass basis. However, in this study, the SLs analyzed were dried, and it was determined that there would be a large difference depending on the variety. The protein and carbohydrate content was high in the proximate composition analysis of *Moringa Oleifera* leaf and *Carica papaya* leaf [18,19]. This demonstrates the potential for ethephon-treated SLs to be used as a good energy and protein source.

Compared to carbohydrates and unsaturated fatty acids, some saturated fatty acids increased, such as total cholesterol, low-density lipoprotein cholesterol, and small high-density lipoprotein cholesterol. High exposure to lauric acid, myristic acid, and palmitic acid may lead to cardiovascular disease and type 2 diabetes mellitus [20]. The primary unsaturated fatty acids are oleic, linoleic, and α-linolenic acids. In particular, linoleic acid and α-linolenic acid are essential fatty acids that cannot be synthesized in the human body and must be consumed [20,21]. Essential fatty acids are positively correlated with cardiovascular disease reduction, diabetes, and high blood pressure through mechanisms including cell membrane composition changes and gene expression [22]. When treating plant hormones in Cabernet Sauvignon grape, most fatty acids increased. However, the trend depended on the treatment concentration, even in SLs treated with ethephon [23]. Saturated fatty acids such as palmitic acid decreased, and α-linolenic acid, an unsaturated fatty acid, increased. Thus, SLs treated with ethephon have great potential as a functional material for health benefits.

Amino acids are constituent units of peptides and proteins, which are the basis of various physiological pathways, activities, and life in general. For example, phenylalanine plays a vital role in the biosynthesis pathway of isoflavone, a secondary metabolite [24]. γ-aminobutyric acid (GABA) is involved in plant growth and development. Thus, proper intake is essential for human health, maintaining blood pressure, preventing aging, and improving liver and kidney function [25]. Studies have shown that when *Torreya grandis* nuts are treated with ethephon, the expression of amino acid biosynthesis genes is upregulated, and degradation enzymes are inhibited simultaneously, increasing amino acid levels [26]. Changes in free amino acids were also observed in ethylene-treated SLs; EAA increased 5 fold and NEAA 6 fold. Thus, ethylene influenced signal transmission and recognition pathways in the growth stage of SLs [27]. This was confirmed by amino acid analysis of ethephon-treated SLs. Although they could not confirm a dramatic increase in free amino acids as in previous studies, they increased with the treatment concentration. Treating growth regulators such as ethephon and ethylene can be an alternative way to increase amino acids with various types and functions in crop cultivation.

Vitamins are essential nutrients for controlling metabolism and body function, and most of them cannot be synthesized in the body, so they should be supplemented through dietary intake [28]. Water-soluble vitamins are composed of vitamin B complex and vitamin C, are involved in many biochemical reactions in the body, and play a role in improving antioxidants and immune responses [29]. Vitamin B5 cooperates with coenzyme A (CoA), which is involved in carbohydrate, fat, and protein metabolism and the Synthesis of acyl carrier proteins [28,29,30]. Vitamin B5 is widely distributed in food and synthesized by intestinal microorganisms, so deficiency rarely occurs in people with a regular diet [29]. Vitamin C is a well-known antioxidant and helps prevent viral infections, reduce folic acid deficiency, and prevent cardiovascular disease. If vitamin C is deficient, scurvy may appear due to inhibition of collagen synthesis [29]. Vitamin C can increase within plants when treated with plant hormones and growth regulators such as cytokinin, gibberellin, and ethylene, depending on the type of crop and fruit [31]. The vitamin C within SL increased significantly through the ethephon, indicating that it can be a good source of the vitamin.

Polyphenols contain phenolic acids, polyphenols, and flavonoids, and phenolic compounds found in plants. Phenol compounds contain various substances, such as anthocyanin, isoflavone, and quercetin, which protect plants and fruits from oxidative damage and are used as antioxidants in humans [32]. TP and TF contents increased in ethylene-treated SLs, which was consistent with the results of this study [13]. The phenolic content increased when ethylene was treated in pitaya fruit. Ethylene increased phenol biosynthesis by inducing critical enzyme activity and gene expression in the phenylpropanoid pathway [33]. TP and TF contents are correlated with antioxidant capabilities, and an increase in phenol compounds according to the ethephon treatment concentration can be expected to increase antioxidant activity [34].

Isoflavone is a plant estrogen flavonoid compound found in soybeans among legumes and is present as a glycoside bound to sugar molecules. Isoflavone benefits menopause, breast cancer prevention, intestinal health improvement, and bone health [35,36,37]. SLs are one of the highest isoflavone concentrations among the soybean plant [7]. Plant hormones, called plant growth regulators, regulate plant growth and are classified as plant growth stimulators and inhibitors according to the mechanism of action. Ethylene acts as a growth promoter and inhibitor depending on the environment [38]. Significant changes in antioxidant activity and metabolite were observed when ethylene was treated in kiwi, and isoflavones increased in ethephon that promotes ethylene generation and SLs treated with ethylene [13,39,40]. This showed a similar trend to the results of this study. Isoflavone synthase induced more by ethephon treatment may have affected isoflavone biosynthesis, and myeloblastosis family transcriptive factors are believed to have accumulated isoflavone by stimulating the transcription of chalcone synthase 7 and chalcone synthase 8 genes essential in isoflavone biosynthesis [13]. The high isoflavone SLs produced by treating plant hormones can be used as functional food materials. To make this efficient, treating ethephon at a more diverse concentration is necessary to check the concentration at which isoflavone reaches its maximum values.

Oxidative stress degrades normal biological function due to free radicals, causes inflammation cardiovascular and metabolic disorders, and can cause cancer if it worsens [41]. Antioxidants are the removal of free radicals to suppress or alleviate the progress of oxidation. Measuring and identifying increased antioxidant activity can bring various health benefits. Among the different parts of soybeans, SLs showed high antioxidant activity, and phenol compounds such as isoflavones were believed to have an effect [7]. After ethylene treatment, antioxidant capacity was improved in lotus roots during the initial storage period, and antioxidant activity was increased and maintained in kiwi through ethylene treatment [39,42]. Ethylene produced by ethephon increases glutathione content and antioxidant metabolism, and the initial rise in ethylene causes an antioxidant effect as a signal response to initiate plant defense [11]. Ethephon produces ethylene under certain environmental conditions above pH 4. The resulting ethylene upregulates certain genes, such as chalcone synthase and isoflavone synthase, in the phenylpropanoid pathway, increasing the production of phenol and flavonoid compounds. The resulting phenolic and flavonoid compounds have many phenolic hydroxyl groups, showing strong antioxidant effects [13]. The increase in isoflavones and phenol compounds was confirmed according to the ethephon treatment concentration in SLs, and the antioxidant activity also showed the same tendency. Phenol compounds are known to be correlated with antioxidant activity, and, specifically, isoflavones contained in soybeans are effective in antioxidant activity [34].

Digestive enzymes include proteolytic enzymes (pepsin, peptidase), carbohydrate-degrading enzymes (amylase, glycosidase), and lipolytic enzymes (lipase). Diabetes is a metabolic disorder caused by high blood sugar and is one of the most common diseases worldwide. Diabetes can be prevented by inhibiting the action of carbohydrate-degrading enzymes and delaying the digestion and absorption of glucose [43]. Obesity is a state of excessive fat tissue in the body, a significant public health problem worldwide, and is highly related to various metabolic diseases. Suppressing the action of lipolytic enzymes can prevent obesity by inhibiting the decomposition of fat and absorption in the body [44]. Anti-obesity and anti-diabetes effects can be seen by inhibiting natural digestive enzymes such as α-amylase and lipase. When soybean’s digestive enzyme inhibition activity was confirmed by growth times, it was observed that the move was high in the roots and leaves and increased by growth times [45]. This study demonstrated that the digestive enzyme inhibition activity of SLs increased according to the ethephon treatment concentration. It shows that SLs treated with ethephon can be used as a functional food material that helps with anti-obesity and diabetes than ordinary SLs.

## 4. Materials and Methods

### 4.1. Materials, Reagents, and Instruments

The SLs used in this study were grown for 60 days after sowing *Daewon* beans (R3 growth stage: maximum growth period before pods are formed) and moved to an air-blocked chamber for ethephon treatment. Ethephon treatment concentrations were 0, 150, and 300 μg/mL, and solid ethephon (MB-E5360, MB Cell Co., Los Angeles, CA, USA) was used dissolved in distilled water. The SLs were treated twice every 24 h interval, and theoretically, the amount of ethylene released by treatment with ethephon at concentrations of 150 and 300 μg/mL is 29.12 and 58.23 μg/mL, respectively. After the final harvest, SLs were collected, washed clean with water, and dried at 35 °C using a hot air dryer (JS-OV-100, JOHNSAM Co., Boocheon, Republic of Korea). After, it was crushed with a food grinder and stored and used at −40 °C (MDF-U5412, Panasonic Co., Osaka, Japan).

Isoflavones (including daidzin, genistin, daidzein, genistein, malonyldaidzin, and malonylgenistin) and reagents used for antioxidant activity, DPPH, ABTS, and 2,4,6-tri(2-pyridyl)-1,3,5-triazine (TPTZ), and reagents used for digestive enzyme inhibitory activity, *p*-nitrophenyl-α-D-glucopyranoside (*p*-NPG), *p*-nitrophenyl-butyrate (*p*-NPB), α-glucosidase, and pancreatic lipase, were purchased from Sigma-Aldrich (Saint Louis, MO, USA). Methanol, acetonitrile, water, and acetic acid were purchased from J.T. Baker (Phillipsburg, NJ, USA). Other reagents were class 1 or special reagents for analysis and purchased and used as needed.

The analysis of free amino acids was performed using an automatic amino acid analyzer (L-8900, Hitachi High-Technologies Co., Tokyo, Japan), and the radical scavenging and digestive enzyme inhibition activity measurement was performed using a spectrophotometer (UV-1800 240V, Shimadzu Corp., Kyoto, Japan). The isoflavones analysis was performed using high-performance liquid chromatography (HPLC, Agilent 1200 system, Agilent Technologies Inc., Waldbronn, Germany). The fatty acids analysis was performed using gas chromatography (GC, Agilent 7890A system, Agilent Technologies Inc., Wilmington, DE, USA).

### 4.2. Analysis of Proximate Compositions

The SL proximate composition content, including moisture, ash, crude fat, carbohydrates, and crude protein, was analyzed by slightly modifying the method outlined by Parimelazhagan et al. [46]. The moisture content was determined by heating at atmospheric pressure, and the ash content was determined by heating in the vacuum furnace at 550 °C. The crude fat content was measured using the Soxhlet method with diethyl ether after grinding the sample, and the crude protein content was measured by the Kjeldahl method. The carbohydrate content was calculated by subtracting the sum of each component from 100% of the total.

### 4.3. Analysis of Fatty Acids

The fatty acid content analysis was performed with a slight modification to the method described by Lee et al. [47]. Fatty acid pretreatment was performed by adding 3 mL of 0.5 N methanolic NaOH to 1 g of the sample, weighing it in a test tube, and heating it at 100 °C for 10 min to perform the hydrolysis of fatty acids and glycerol. Then, 2 mL of boron trifluoride (BF_3_) was added and stirred, followed by heating for another 30 min to carry out the methyl esterification of fatty acids. After the completion of the methyl esterification reaction, 1 mL of iso-octane was added, vigorously shaken, and left to stand. Only the iso-octane layer was collected, dehydrated with anhydrous sodium sulfate, and filtered through a 0.45 μm membrane filter (Dismic-25CS, Toyoroshikaisha, Ltd., Tokyo, Japan) before analysis by GC. Nitrogen gas was used as the moving phase, and the moving phase flow rate was maintained at 1 mL/min. The oven temperature was initially raised to 140 °C and held for 5 min, then increased at a rate of 20 °C per min to 180 °C and held for 2 min. Then, the temperature increased in increments of 5 °C to 230 °C and held for a final 35 min. The injector and flame ionization detector temperatures were set at 220 °C and 240 °C, respectively.

### 4.4. Analysis of Free Amino Acids

Free amino acids were analyzed in SLs using Lee et al.’s [48] method. Next, 1 g of the sample was homogenized with 5 mL of distilled water and then hydrolyzed at 60 °C for 1 h using a heat block (HB-48P, DAIHAN Scientific, Wonju, Republic of Korea). After hydrolysis, 1 mL of 10% 5-sulfosalicylic acid dihydrate was added, mixed, left at 4 °C for 2 h, and then centrifuged at 15,000 rpm for 3 min and filtered with a syringe filter. The filtrate was depressurized and concentrated at 50 °C using a rotary evaporator. Then, 2 mL of lithium buffer (pH 2.2) was added for dissolution. The solution was filtered through a 0.45 μm membrane filter and quantitatively analyzed using an automatic amino acid analyzer.

### 4.5. Analysis of Water-Soluble Vitamin

Water-soluble vitamin analysis was performed by modifying the Datta et al. [49] method. The extract was prepared by extracting the SLs powder sample 1 g with 50% MeOH 10 mL for 12 h, centrifuging at 4000 rpm for 30 min, and filtering the supernatant through a 0.45 µm-membrane filter. The filtrate was analyzed using HPLC, on a mobile phase A analysis column using 0.2% acetic acid in HPLC water, mobile phase B using 0.2% acetic acid in acetonitrile, and a LiChrospher^®^ 100 RP C18 column (4.6 × 250 mm, 5 µm, Merck KGaA, Darmstadt, Germany). The sample injection was 20 mL, the flow rate was 1 mL/min, the column temperature was 30 °C, and the analysis wavelength was 256 nm. The linear gradients of solvent B were 0% (0 to 5 min), 0 to 75% (5 to 15 min), and 75% (15 to 25 min).

### 4.6. Preparation of Extracts

SLs were extracted at room temperature for 12 h by adding 20 fold of 50% MeOH to 1 g of SL powder. The extract was centrifuged for 30 min. The supernatant was filtered with a 0.45 μm membrane filter to measure phenolics, flavonoids, and isoflavones content and physiological activity.

### 4.7. Analysis of Total Phenolic and Flavonoid Contents

The TP content was measured by slightly modifying the method detailed by Pungin et al. [50]. Next, 0.5 mL of the extract was distributed in a test tube, and 0.5 mL of a 25% Na_2_CO_3_ solution was added and incubated for 3 min. Then, 0.25 mL of the 2 N Folin-Ciocalteu phenol reagent was added, combined, and then incubated at 30 °C for 1 h to allow the color to develop. The blue color was measured using a spectrophotometer at 750 nm. At this time, the TP content was obtained from the standard curve prepared using gallic acid. Each experiment was repeated three times and expressed as an average value.

The TF content was measured by slightly modifying the Pungin et al. [50] method. 0.5 mL of the diluted extract was aliquoted in a test tube, and 1 mL of diethylene glycol and 0.01 mL of 1 N-NaOH were added. It was incubated in a 37 °C constant-temperature bath for 1 h, and absorbance was measured at 420 nm using a spectrophotometer. The TF content was obtained from the standard curve prepared using rutin. Each experiment was repeated three times and expressed as an average value.

### 4.8. Analysis of Isoflavones

The isoflavones analysis was performed by HPLC following the Lee et al. [48] method. The Lichrophore 100 RP C18 columns were used for the analysis (LichroCART 125–4, 5 µm, 125 mm × 4 mm, Merck KGaA, Darmstadt, Germany). The moving phase solvent was analyzed as 0.2% glacial acetic acid in water (solution A) and 100% acetonitrile (solution B). The moving phase was analyzed at 0 min–100%, 15 min–90%, 25 min–80%, 30 min–75%, 45 min–65%, and 50 min–65% based on the A solvent. The sample was injected with 20 μL, and the moving phase flow rate was maintained at 1 mL/min at 30 °C. A diode array detector was used as the detector, and quantification was performed at a 254 nm absorbance. Each experiment was repeated three times and is expressed as an average value. The HPLC chromatogram of six isoflavone derivatives and standard substances is shown in Figure 5A.

### 4.9. Analysis of Antioxidant Activity

Antioxidant activity was measured by slightly modifying the Lee et al. [48] method. Briefly, DPPH radical scavenging activity was measured in a mixture of 0.8 mL of 1.5 × 10^−4^ M DPPH solution, and 0.2 mL of the extracted sample was homogenized for 10 s and incubated in the dark for 30 min. The absorbance was measured at 525 nm using a spectrophotometer. ABTS radical scavenging activity was measured in 7 mM ABTS solution (2.5 mL) and 2.45 mM K_2_S_2_O_8_ (7.5 mL) mixed and left in a darkroom for 14 ± 2 h. Then, it was mixed with methanol and used as an ABTS radical solution with the absorbance value of the control group adjusted to 0.7 ± 0.02 at 732 nm. Next, 0.1 mL of the diluted sample was mixed with 0.9 mL of the ABTS radical solution and incubated in the dark for 3 min. Absorbance was measured at 732 nm. The hydroxyl (OH) radical scavenging activity solution was 0.2 mL of a 10 mM FeSO_4_∙7H_2_O-EDTA solution mixed with 1.4 mL of the sample. It was incubated at 37 °C for 4 h, and 1 mL of 1% thiobarbituric acid (TBA) and 2.5% trichloroacetic acid (TCA) was mixed and heated in boiling water at 100 °C for 10 min to add color. The colored sample was cooled at 25 °C, and the absorbance was measured at 520 nm in a spectrophotometer. In the DPPH and ABTS radical scavenging activity assays, an extraction solvent was used instead of a sample for the negative control, and phosphate buffer saline was used instead of a sample for the hydroxyl (OH) radical scavenging activity. The absorbance of the experiment and negative control was measured and expressed as a percentage (%), as shown in the following Equation (1):

Radical scavenging activity (%) = [1 − (negative control absorbance/experimental control absorbance)] × 100(1)

FRAP assay was prepared by mixing 300 mM acetate buffer (pH 3.6), 10 mM TPTZ reagent, and 20 mM FeCl_3_ solution at 10:1:1 (*v*/*v*/*v*). The mixed solution was used after a preliminary reaction for 15 min in a 37 °C water bath. 0.05 mL of sample and 0.95 mL of FRAP reagent were mixed and incubated at 37 °C for 15 min, and absorbance was measured at 590 nm using a spectrophotometer.

### 4.10. Analysis of Digestive Enzymes Inhibitory Activity

The digestive enzyme inhibitory activity was measured by slightly modifying the Hwang et al. [51] method. For α-glucosidase and pancreatic lipase inhibitory activities, 0.03 mL of appropriately diluted sample was placed in a test tube with 0.07 mL of a 1.0 U/mL α-glucosidase and pancreatic lipase enzyme solution and 0.05 mL of a 200 mM sodium phosphate buffer (pH 6.8). Next, 0.1 mL of 10 mM *p*-NPG and *p*-NPB were dissolved in the buffer and added to the mixtures. The reactions were performed at 37 °C for 10 min. Finally, 0.75 mL of 100 mM Na_2_CO_3_ was added to quench the reaction, and the absorbance was measured at 420 nm. The inhibition rate is expressed as a percentage by the following Formula (2):

(2) $$\mathrm{Digestive}\;\mathrm{enzyme}\;\mathrm{Inhibition}\;\mathrm{activity}\left(\%\right)=[1-(\mathrm{negative}\;\mathrm{negative}\;\mathrm{control}\;\mathrm{absorbance}/\mathrm{experimental}\;\mathrm{control}\;\mathrm{absorbance})]\times 100$$

### 4.11. Statistical Analysis

In all experiments, negative control experiments were performed with extractive solvents instead of samples, measurements were repeated three times, and the experimental results were expressed as mean ± standard deviation. Statistical analyses included the ANOVA procedure, followed by Duncan’s multiple range test (*p* < 0.05). All statistical analyses were performed using the Statistical Analysis System (SAS) software. Statistical version 9.4 (SAS Institute, Cary, NC, USA). Nutritional components and isoflavone data were visualized as a heatmap using Multiple Experiment Viewer software (MeV 4.9.0) [52].

## 5. Conclusions

This research confirmed the metabolic changes in SLs treated with the chemical inducer ethylene and its precursor, ethephon. Varying concentrations of treatment to investigate the SLs proximate compositions, nutritional components (such as fatty acids, free amino acids, and water-soluble vitamins), antioxidant activities (including DPPH, ABTS, and hydroxyl radical scavenging activities and FRAP), enzyme inhibition activities (such as α-glucosidase and lipase), and secondary metabolites (TP, TF, and Isoflavone contents). There were no significant differences observed in proximate compositions and fatty acids. However, free amino acids increased with treatment concentrations, particularly GABA, which increased slightly in NEAA, and phenylalanine grew about 2 fold in EAA. Antioxidant and enzyme inhibition activities increased with increasing ethephon treatment concentration, except for hydroxyl radical. The TP and TF contents increased in secondary metabolites according to the ethephon treatment concentration. Isoflavone changed significantly, growing 5 to 10 fold depending on the component and treatment concentrations. These results indicate that primary and secondary metabolites increase depending on the ethephon treatment concentration. Based on these research findings, SL production efficiency and utilization value could be enhanced if applied in various plant cultivation systems under rapidly changing environments such as climate change.

## Figures and Tables

**Table 1:** Comparison of proximate composition in soy leaves by ethephon treatment.

Contents ^1^ (g/100 g)	Treatment Concentration of Ethephon ^2^ (μg/mL)		
	SL1	SL2	SL3
Moisture	9.00 ± 0.54 ^a^	8.90 ± 0.53 ^a^	8.60 ± 0.52 ^a^
Ash	9.90 ± 0.59 ^a^	8.80 ± 0.53 ^b^	10.00 ± 0.61 ^a^
Protein	20.60 ± 1.24 ^b^	20.90 ± 1.25 ^b^	25.00 ± 1.50 ^a^
Fat	3.30 ± 0.20 ^b^	5.30 ± 0.32 ^a^	3.40 ± 0.19 ^b^
Carbohydrate	57.20 ± 3.43 ^a^	56.10 ± 3.37 ^ab^	53.00 ± 3.18 ^b^

^1^ All values are presented as the mean ± SD of triplicate determination. Means with different letters within a row are significantly different between samples for the same index (*p* < 0.05). ^2^ Ethephon treatments at 0 μg/mL, SL1; Ethephon treatments at 150 μg/mL, SL2; and Ethephon treatments at 300 μg/mL, SL3.

**Table 2:** Comparison of water-soluble vitamin contents in soy leaves by ethephon treatment.

Contents ^1^ (mg/100 g)	Treatment Concentration of Ethephon ^3^ (μg/mL)		
	SL1	SL2	SL3
B2 (Riboflavin)	0.10 ± 0.01 ^a^	nd ^2^	0.10 ± 0.01 ^a^
B3 (Niacin)	21.90 ± 1.31 ^a^	8.80 ± 0.53 ^c^	14.30 ± 0.86 ^b^
B5 (Pantothenic acid)	215.70 ± 12.94 ^b^	206.00 ± 12.36 ^b^	431.00 ± 25.86 ^a^
B9 (Folic acid)	8.80 ± 0.53 ^c^	12.30 ± 0.74 ^b^	15.40 ± 0.92 ^a^
C (Ascorbic acid)	0.20 ± 0.01 ^c^	17.60 ± 1.06 ^b^	40.80 ± 2.45 ^a^
Total	246.70	244.70	501.60

^1^ All values are presented as the mean ± SD of triplicate determination. Means with different letters within a row are significantly different between samples for the same index (*p* < 0.05). ^2^ nd: not detected. ^3^ Ethephon treatments at 0 μg/mL, SL1; ethephon treatments at 150 μg/mL, SL2; ethephon treatments at 300 μg/mL, SL3.

**Table 3:** Comparison of isoflavone contents in soy leaves by ethephon treatment.

Contents ^1^ (μg/g)	Treatment Concentration of Ethephon ^2^ (μg/mL)		
	SL1	SL2	SL3
Glycosides			
Daidzin (1)	279.08 ± 18.01 ^c^	1652.33 ± 99.14 ^b^	2076.14 ± 124.57 ^a^
Genistin (2)	216.10 ± 12.97 ^c^	831.35 ± 49.88 ^b^	1664.39 ± 99.86 ^a^
Total	495.18	2483.67	3740.54
Malonylglycosides			
Daidzin (3)	354.12 ± 21.25 ^c^	3264.16 ± 195.85 ^b^	5673.07 ± 340.38 ^a^
Genistin (4)	505.07 ± 30.30 ^c^	1896.63 ± 113.80 ^b^	5238.65 ± 314.32 ^a^
Total	859.20	5160.79	10,911.73
Aglycones			
Daidzein (5)	33.58 ± 2.01 ^c^	117.23 ± 7.03 ^b^	208.62 ± 12.52 ^a^
Genistein (6)	42.15 ± 2.53 ^b^	44.73 ± 2.68 ^b^	107.11 ± 6.43 ^a^
Total	75.73	161.96	315.73
Total isoflavones	1430.11	7806.42	14,968.00

^1^ All values are presented as the mean ± SD of triplicate determination. Means with different letters within a row are significantly different between samples for the same index (*p* < 0.05). ^2^ Ethephon treatments at 0 μg/mL, SL1; ethephon treatments at 150 μg/mL, SL2; ethephon treatments at 300 μg/mL, SL3.

## Data Availability

All the data are available in the paper.

## Supplementary Materials

The following supporting information can be downloaded at: https://www.mdpi.com/article/10.3390/plants12203640/s1, Table S1: comparison of fatty acid contents in soybean leaves by ethephon treatment; Table S2: comparison of free amino acid contents in soy leaves by ethephon treatment.
