# Supplementary material for: Changes in Nutrient Components and Digestive Enzymatic Inhibition Activities in Soy Leaves by Ethephon Treatment
Source: Plants (Basel). 2023 Oct 21;12(20):3640. doi: 10.3390/plants12203640 (PMC10609866; doi:10.3390/plants12203640)
Supplement: Supplementary file 1 [file plants-12-03640-s001.zip › plants-2629564-supplementary.pdf]

Table S1. Comparison of fatty acid contents in soybean leaves by ethephon treatment

Contents [1] (mg/100 g)	Treatment concentration of ethephon [3] (μg/mL)		
	SL1	SL2	SL3
Saturated fatty acids (SFA)			
Palmitic acid (C16:0)	212.30 ± 10.62 [a]	224.80 ± 16.74 [a]	191.20 ± 9.56 [b]
Stearic acid (C18:0)	65.70 ± 3.29 [b]	70.50 ± 4.03 [a]	52.70 ± 2.64 [c]
Arachidic acid (C20:0)	4.80 ± 0.24 [b]	5.30 ± 0.22 [a]	5.00 ± 0.25 [b]
Behenic aicd (C22:0)	6.50 ± 0.33 [c]	8.50 ± 0.38 [a]	7.30 ± 0.37 [b]
Lignoceric acid (C24:0)	8.40 ± 0.42 [c]	10.70 ± 0.44 [a]	10.00 ± 0.50 [b]
Total	297.70	319.80	266.20
Unsaturated fatty acids (USFA)			
Palmitoleic acid (C16:1)	31.50 ± 1.57 [a]	32.00 ± 1.60 [a]	21.10 ± 1.06 [b]
Oleic acid (C18:1n9c)	90.00 ± 4.5 [ab]	87.30 ± 3.37 [b]	93.70 ± 4.69 [a]
Linoleic acid (C18:2n6c)	163.50 ± 8.18 [a]	167.40 ± 13.37 [a]	161.50 ± 8.08 [a]
γ-Linolenic acid (C18:3n6)	3.10 ± 0.16 [a]	nd [2]	nd
α-Linolenic acid (C18:3n3)	383.80 ± 19.19 [b]	419.60 ± 40.98 [a]	447.10 ± 22.36 [a]
Eicosenic acid (C20:1)	2.70 ± 0.14 [b]	4.10 ± 0.21 [a]	1.70 ± 0.09 [c]
Eicosatrienoic acid (C20:3n3)	1.90 ± 0.10 [c]	3.80 ± 0.19 [a]	2.90 ± 0.15 [b]
Nervonic acid (C24:1)	2.70 ± 0.14 [b]	3.70 ± 0.39 [a]	2.50 ± 0.13 [c]
Total	679.20	717.90	730.50
Total fatty acids	976.90	1037.70	996.70

[1] All values are presented as the mean±SD of triplicate determination. Means with different letters within a row are significantly different between sample for the same index ($p < 0.05$)

[2] nd: not detected.

[3] Ethephon treatments at 0 μg/mL, SL1; Ethephon treatments at 150 μg/mL, SL2; Ethephon treatments at 300 μg/mL, SL3.

Table S2. Comparison of free amino acid contents in soy leaves by ethephon treatment

Contents [1] (mg/100 g)	Treatment concentration of ethephon [3] (μg/mL)		
	SL1	SL2	SL3
Non-essential amino acids (NEAA)			
Taurine	7.20 ± 0.43 [c]	8.40 ± 0.50 [b]	9.20 ± 0.55 [a]
Urea	79.40 ± 4.76 [c]	158.00 ± 9.48 [b]	304.40 ± 18.26 [a]
Aspartic acid	174.20 ± 10.45 [b]	179.80 ± 10.79 [ab]	189.60 ± 11.38 [a]
Serine	69.20 ± 4.15 [c]	79.20 ± 4.75 [b]	132.80 ± 7.97 [a]
Glutamic acid	65.80 ± 3.95 [c]	88.00 ± 5.25 [b]	132.80 ± 7.97 [a]
Sarcosine	nd [2]	2.40 ± 0.14 [a]	nd
Aminoadipic acid	32.80 ± 1.97 [c]	42.80 ± 2.57 [b]	57.20 ± 3.34 [a]
Glycine	14.60 ± 0.88 [c]	20.60 ± 1.24 [a]	19.40 ± 1.16 [a]
Alanine	116.80 ± 7.01 [a]	90.60 ± 5.44 [c]	108.60 ± 6.52 [b]
Citrulline	3.60 ± 0.22 [b]	6.20 ± 0.37 [a]	6.20 ± 0.37 [a]
α-aminobutyric acid	6.80 ± 0.41 [c]	9.00 ± 0.54 [b]	10.40 ± 0.62 [a]
Cystathionine	4.40 ± 0.26 [c]	6.60 ± 0.40 [a]	5.00 ± 0.30 [b]
Tyrosine	52.80 ± 3.17 [a]	29.20 ± 1.75 [c]	45.80 ± 2.75 [b]
β-alanine	21.60 ± 1.30 [a]	22.80 ± 1.37 [a]	18.20 ± 1.09 [b]
β-aminoisobutyric acid	18.00±1.08	23.40 ± 1.40 [a]	nd
γ-aminobutyric acid	141.00±8.46 [b]	182.20 ± 10.93 [a]	182.60 ± 10.96 [a]
Aminoethanol	8.80±0.53 [b]	7.20 ± 0.43 [c]	10.40 ± 0.62 [a]
Hydroxyproline	0.80±0.05 [c]	1.40 ± 0.08 [a]	1.00 ± 0.06 [b]
Ornithine	1.40±0.08 [c]	1.60 ± 0.10 [b]	2.60 ± 0.16 [a]
1-Methylhistidine	3.20±0.19 [b]	5.20 ± 0.31 [a]	3.40 ± 0.20 [b]
Arginine	141.00±8.46 [b]	82.40 ± 4.94 [c]	192.00 ± 11.52 [a]
Total	963.40	1,047.00	1,431.60
Essential amino acids (EAA)			
Threonine	30.40 ± 1.82 [c]	58.60 ± 3.52 [a]	52.40 ± 3.14 [b]
Valine	115.80 ± 6.95 [c]	126.20 ± 7.57 [b]	166.40 ± 9.98 [a]
Methionine	11.20 ± 0.67 [b]	12.40 ± 0.74 [a]	11.00 ± 0.66 [b]
Isoleucine	62.20 ± 3.37 [c]	71.40 ± 4.28 [b]	82.60 ± 4.96 [a]
Leucine	61.40 ± 3.68 [b]	93.00 ± 5.58 [a]	95.60 ± 5.74 [a]
Phenylalanine	84.00 ± 5.04 [c]	108.60 ± 6.52 [b]	157.00 ± 9.42 [a]
Lysine	58.20 ± 3.49 [b]	33.20 ± 1.99 [c]	68.80 ± 4.13 [a]
Histidine	26.40 ± 1.58 [b]	19.40 ± 1.16 [c]	35.00 ± 2.10 [a]
Total	449.60	522.80	668.80
Total amino acids	1413.00	1569.80	2100.40

[1] All values are presented as the mean±SD of triplicate determination. Means with different letters within a row are significantly different between sample for the same index ($p < 0.05$)

[2] nd: not detected.

[3] Ethephon treatments at 0 μg/mL, SL1; Ethephon treatments at 150 μg/mL, SL2; Ethephon treatments at 300 μg/mL, SL3.
